# Engineered Aptamers to Probe Molecular Interactions on the Cell Surface

**DOI:** 10.3390/biomedicines5030054

**Published:** 2017-08-29

**Authors:** Sana Batool, Sanam Bhandari, Shanell George, Precious Okeoma, Nabeela Van, Hazan E. Zümrüt, Prabodhika Mallikaratchy

**Affiliations:** 1 Department of Chemistry, Lehman College, The City University of New York, 250 Bedford Park Blvd. West, Bronx, New York, NY 10468, USA; Sana.Batool@lc.cuny.edu (S.B.); sanam.bhandari@lc.cuny.edu (S.B.); Shanell.George2@lc.cuny.edu (S.G.); preciousokeoma@hotmail.com (P.O.); Nabeela.Van@lc.cuny.edu (N.V.); 2 Ph.D. Programs in Chemistry and Biochemistry, CUNY Graduate Center, 365 Fifth Avenue, New York, NY 10016, USA; hzumrut@gradcenter.cuny.edu; 3 Ph.D. Program in Molecular, Cellular and Developmental Biology, CUNY Graduate Center, 365 Fifth Avenue, New York, NY 10016, USA

**Keywords:** aptamers, nanosensors, nanorobots, molecular modulators, therapeutics, diagnostics

## Abstract

Significant progress has been made in understanding the nature of molecular interactions on the cell membrane. To decipher such interactions, molecular scaffolds can be engineered as a tool to modulate these events as they occur on the cell membrane. To guarantee reliability, scaffolds that function as modulators of cell membrane events must be coupled to a targeting moiety with superior chemical versatility. In this regard, nucleic acid aptamers are a suitable class of targeting moieties. Aptamers are inherently chemical in nature, allowing extensive site-specific chemical modification to engineer sensing molecules. Aptamers can be easily selected using a simple laboratory-based in vitro evolution method enabling the design and development of aptamer-based functional molecular scaffolds against wide range of cell surface molecules. This article reviews the application of aptamers as monitors and modulators of molecular interactions on the mammalian cell surface with the aim of increasing our understanding of cell-surface receptor response to external stimuli. The information gained from these types of studies could eventually prove useful in engineering improved medical diagnostics and therapeutics.

## 1. Introduction

More than 50 years ago, researchers began to speculate that cell surface molecules respond to external stimuli and perhaps undergo modification at the membrane level [1]. Indeed, considerable empirical evidence over the years has demonstrated that the cell surface does undergo structural changes at the molecular level in response to either external or internal stimuli and that such changes lead to alterations in cell signaling and transforming the function of a cell [2,3,4]. How this altered expression of molecules lead to modifications of a cell’s membrane structure remains to be elucidated [5]. While one third of the genome encodes membrane receptors compared to soluble proteins, only a few atomic structures of membrane protein structures have been identified [6,7,8]. All of these structures were solved in their purified form, not in their native form on the cell surface [6,7,8]. Furthermore, out of known membrane protein receptors, many are for bacterial proteins. These challenges have further diminished the progress in deciphering molecular interactions that take place on the cell membrane.

Elucidating the function of cell membrane protein receptors is important, as the cell membrane is the first structure to make contact with external stimuli. For example, it is the cell’s receptors that recognize invading pathogens and signal the nucleus for response [4,9]. As noted, it is not unusual for cell surface receptors to undergo a series of conformational changes induced by binding of molecular entities during these recognition events [10]. It has been shown that specific bacterial pathogens target surface proteins, frequently identifying integrin, cadherin, and immunoglobulin-related cell adhesion molecules on host cells and tissues [11]. However, we still do not know enough about the resultant changes at the molecular level in the host cell, knowledge that would otherwise allow us to generate new classes of molecules to address antibacterial resistance. Furthermore, while the mechanism of toxicity is substantially different in the innate and adaptive immune responses, initial events in both responses are begun by molecular interactions with the immune cell or host cell surfaces [12,13]. Yet, the engineering of molecular tools to elucidate and modulate these interactions remains poorly investigated. Thus, it can be convincingly concluded that the chemistry and biology of cell membrane protein receptors, as they interact with external molecular entities in their native state, remain largely uninvestigated.

We have only limited structural knowledge about membrane proteins. Interestingly, however, owing to the unique function of the membrane protein receptors, most drugs are designed against cellular receptors. In addition, most immunotherapeutic molecules target molecular interactions on the cell surface, even when knowledge about such interactions is yet to be discovered in detail [14,15]. Most diagnostic molecules used in disease detection are also mainly aimed at membrane proteins [16,17]. For example, nearly all hematopoietic diseases are detected by examining the expression profiles of receptors, utilizing a panel of antibodies against CD specificities, and molecular imaging agents mainly target membrane proteins [18]. Progress has been made in developing molecules to carry a payload able to detect the differential expression of cell surface markers in response to an external stimulus [19,20]. Molecular tools could be used to sense structural changes on the cell membrane. For example, in addition to changes in expression levels of membrane proteins in response to external stimuli, it has been shown that actin and associated proteins control the regulation of cellular movement, cell-cell interactions and cell-receptor ligand interactions [21]. During this process, the cellular membrane is reoriented, while its structural integrity remains intact [21]. Real-time detection of such events using small synthetic molecular probes could expand our understanding of how cells migrate. Most recently, it has been revealed that all cells secrete extracellular vesicles, which act as transporters of cellular information playing a significant role in cell-cell communication [22]. Development of molecular tools to detect the formation of extracellular vesicles could be significant with potential for development of improved diagnostics.

Nucleic acid aptamers are functional molecules that could be used as a basis for developing molecular tools in detecting the events just discussed. Aptamers are inherently chemical in nature, thus allowing extensive site-specific chemical modification [23,24,25]. They are composed of nucleic acids that can be chemically synthesized and incorporated with diverse chemical functionalities that allow facile detection. Thus, aptamers are operationally less challenging than probes of biological origin, namely antibodies. Most importantly, in the near future, it is fair to speculate that all aptamers might have some level of incorporation of modified nucleic acids to address two key challenges of aptamers, enhancement of nuclease resistance and structural diversity, thus facilitating the generation of aptamers with higher affinity [26,27,28,29,30]. These two key improvements will play a predominant role in promoting aptamers as diagnostic and therapeutic agents. The use of aptamers as diagnostic tools to detect the composition of established biomarker membrane proteins makes them a clinical imperative. It is important to point out that the stability and large-scale production of aptamers is superior to that of antibodies and that this can be advantageous in engineering diagnostic molecules to detect established biomarkers. The lower cost of synthesis and thermostability would also be helpful in using aptamers as sensing molecules [31,32,33,34]. Finally, the intrinsic nature of nucleic acid self-assembly can be effectively utilized in designing molecular tools [35]. Incorporation of fluorophores and other types of signaling strategies will enable detection of events as they happen in real-time on cell membranes [36]. In this short review, we will discuss a few examples in which scaffolds have been engineered with aptamers to detect cell surface protein receptor interactions.

## 2. Engineering Aptamers to Rapidly Sense Pathogens and Infection

Recently, such organizations as the World Health Organization (WHO), Center for Disease Control and prevention (CDC), the Infectious Diseases Society of America, and the World Economic Forum have focused on antibacterial resistance as one of the major challenges in treating infectious diseases [37,38]. The emergence of pathogens with evolved antibiotic resistance requires an immediate solution [38]. Antibacterial resistance and the evolution of bacterial strains are widely reported, and new pathogenic strains are continually detected, highlighting the need for rapid point-of-care screening [39]. Although quick detection of bacteria is important, conventional methods used to diagnose pathogen infection are time-consuming and expensive [40]. As an alternative to advanced instrumentation and technical expertise, molecules that can be utilized in electrochemical or optical sensors are more desirable owing to their lower limit of detection, simplicity, and rapid signal output. While it is imperative to understand the molecular basis of pathogenic interactions, the current trend in aptamer application is predominately focused on the development of aptamer-based diagnostics to detect pathogens. Fittingly, aptamers can be engineered to meet the requirements of robust point-of-care diagnostics for the detection of pathogens. For example, Bruno et al. showed that aptamer-based sensors could be developed to detect variety of foodborne pathogens utilizing colorimetric detection using quantum dots [41]. Furthermore, Chang et al. recently developed a sensing platform based on aptamers conjugated to gold nanoparticles (AuNPs), resulting in a sensing device able to detect *Staphylococcus aureus* [42]. That these AuNPs, when combined with an optical signal, could achieve such rapid and accurate detection shows the promise of aptamers in bacterial detection by means of optical sensing. Marton et al. successfully used high-affinity aptamers for the rapid detection of *E. coli* [43]. The authors showed that these same aptamers could bind to meningitis/sepsis-associated *E. coli* (MNEC) from clinical samples, demonstrating the applicability of aptamers in detecting pathogens in clinical isolates. Tang et al. showed the potential of aptamers in developing known biomarker molecules expressed during an infection [44]. In particular, the antigen mannose-capped lipoarabinomannan (ManLAM) was shown to be released during *Mycobacterium Tuberculosis* (TB) infection. This study shows the development of a highly sensitive and specific aptamer-based diagnostic platform utilizing an aptamer selected against ManLAM [44]. Furthermore, utilizing a modified uracil analogue, whole cell-SELEX was employed to select aptamers against *E. coli* DH5α cells by Renders et al., demonstrating the utility of modified libraries to identify aptamers against pathogens [45]. Detection of viral infection could also be approached utilizing aptamers against virally infected proteins. For example, a multimerized aptamer targeted against Transferrin receptor (TfR), which was intially selected against human transferrin receptor, was also shown to block infection of recombinant New World Hemorrhagic Fever Mammarenaviruses (NWM) in human cells [46]. A report by Lee et al. demonstrated that a truncated aptamer against the hepatitis C virus (HCV) prevented it from replicating [47]. In addition, aptamers were selected and utilized to block the effect of Herpes simplex virus type 2 (HSV-2), HIV proteins, and Dengue virus [48,49,50,51,52,53,54]. A number of studies have shown how an electrochemical and optical sensing platform coupled with the targeting ability of aptamers could be utilized in effectively detecting pathogenic microorganisms, and these have been extensively discussed in a number of reviews [55,56,57].

## 3. Aptamers Engineered to Rapidly Sense Cellular Interactions

To expand our understanding how cell receptors in the immune system and their interactions, to sense altered expression patterns of cell membrane receptors, it is essential to understand and, perhaps, visualize these interactions in real time. However, thus far, the focus has been on monitoring, or mimicking, these interactions by utilizing bulk measurements with a highly heterogeneous population of cells [58]. In order to understand single molecules interacting with each other, it is imperative to develop molecular tools for single-cell analysis. Here, too, aptamers could be utilized to engineer molecular probes able to sense such interactions at the cell surface. The use of self-assembled properties, together with a wide range of synthetic capabilities and known targeting ability, can be effectively integrated to engineer functional scaffolds to control, sense, and stimulate intercellular interactions.

### 3.1. Aptamers as Logic Gates

**Figure 1:**
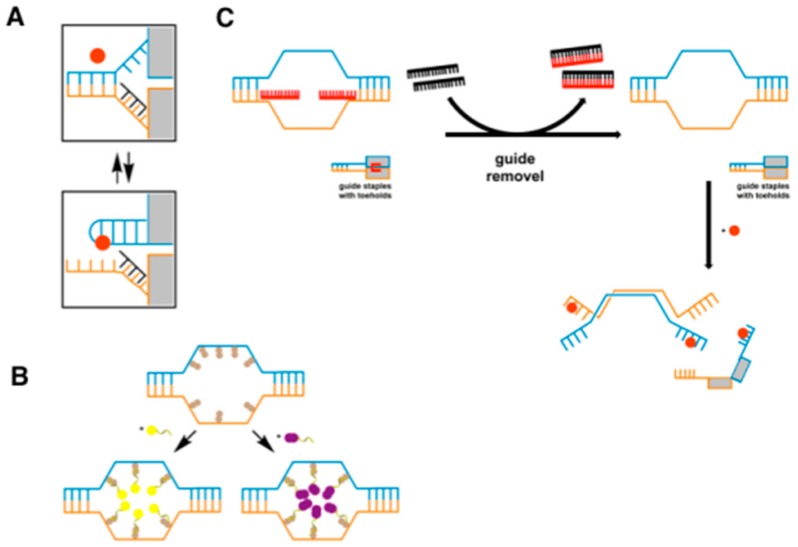
Design of an aptamer-gated DNA nanorobot. (**A**) Aptamer (**blue**) and complementary strand (orange)-based lock mechanisms. The lock dissociates and stabilizes in the presence of the antigen (**red**); (**B**) Nanorobots can be loaded with gold nanoparticles (**yellow**) or antibody Fab′ fragments (**purple**); (**C**) Guide staples in the front and side view of the nanorobots. Multiple eight-base toehold sequences guide the assembly of closed state nanorobots. After folding, addition of the complementary strand removes the guide staples, and the nanrobots can then be activated upon interaction with the antigen [59].

Using aptamers selected against membrane proteins, logic gates have recently been used to sense molecular interactions on the cell membrane. Intriguingly, a self-employed DNA-based nanorobot was constructed with DNA aptamers to target cell surface molecules as a site-selective drug delivery strategy [59]. This DNA origami based nanorobot is designed such that a “DNA box”, which carries the payload, opens up based on the interactions between cell surface molecules and the targeting moiety attached to the DNA box [59]. Thus, drug delivery depends on different molecular signatures on the cell instead of just one surface marker [59]. The authors designed a clasp system based on locks utilizing DNA aptamers, which are designated as “keys” to activate the unloading of a payload in the DNA box, thus, opening of the box exclusively depends on aptamer interaction with its cell-surface target. This study utilized aptamers against platelet-derived growth factor (PDGF), an unknown receptor on B cells, and PTK7 on T and NK cells, and they were engineered to perform the Boolean AND operation as keys. Different nanorobots were constructed to recognize their key antigens expressed on cell lines (Figure 1). When the right combination of antigens is present on the cell membrane, i.e., antigen X AND antigen Y, aptamer/antigen binding unlocks DNA boxes carrying the drug payload. Here is a perfect example of the specific recognition of aptamer to its respective antigen programmed as a logic gate to generate a precise signal output.

Similarly, You et al. showed the incorporation of aptamers with AND, OR, and NOT Boolean logic gates to effectively detect multiple markers expressed on lymphoblastic leukemia cells and lymphoma cells [60]. These types of constructs could only be engineered using aptamer-based molecules because both the payload-containing nanodevice and the targeting moiety could be combined into one molecule without requiring any bioconjugation reactions.

**Figure 2:**
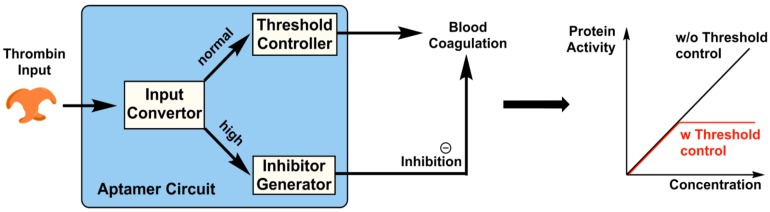
The working principle of aptamer based molecular circuit. The circuit’s three interconnected modules could be programmed to control the activity of the protein [63]. The molecular circuit operated by series of strand-displacement reactions followed by aptamer-protein recognition [63].

A number of follow-up studies demonstrated a broad range of applications for this initial logic gate concept. Taking advantage of the programmability of DNA, together with the ligand recognition of an aptamer via an induced fit mechanism, Yang et al. demonstrated how geometric patterns of self-assembled DNA nanostructures could be controlled and incorporated into nano-devices [61]. In this study, utilizing two small-molecule binding aptamers, one against cocaine and the other against adenosine triphosphate (ATP), in conjunction with DNAzymes, the authors show how DNA origami tiles could be programmed to generate a specific series of OR, YES, and AND logic gates by incorporating aptamer conformational shift in response to small molecules. In a different study, Han et al. demonstrated that sensors could be developed into a prototype biochemical circuit using a DNA aptamer and protein interactions [62]. Here, authors demonstrate a logic gate system using specific binding of two aptamers to human thrombin to construct a molecular circuit. The circuit, which is programmable and autonomous, consists of an input convertor, a threshold controller and an inhibitor generator. Incorporation of these regulatory units to control blood coagulation by thrombin aptamer elegantly demonstrates the applicability of engineered aptamers in the design of regulatory circuits using two aptamers against a target molecule (Figure 2).

The circuit comprised a series of aptamer-binding and strand displacement reactions by introducing a higher affinity recognition molecule, followed by a regulated inhibitory mechanism based on the concentration of DNA input. At high DNA input concentration, the device is designed to change conformation, becoming an inhibitor generator model in which the inhibitor is a second thrombin-binding aptamer able to inhibit its function by binding to exosite II of thrombin (Figure 2). Aptamers and logic gates have also been incorporated into nanomaterials. For instance, Yin et al. showed that aptamers and logic gates could be incorporated into aptamer cross-linked hydrogels to impact their assembly and disassembly to produce an output colorimetric signal [64]. These types of molecular circuits and robots that can sense and control the biological interactions are essential in designing novel and smart drug delivery and sensing strategies.

### 3.2. Aptamers as Immunomodulators

In addition to the development of sensors and diagnostic tools, aptamers have also been designed and engineered to navigate and interpret immune responses. For example, Pastor et al. isolated an aptamer against CD28, which was later engineered into a dimeric version, successfully demonstrating that bivalent anti-CD28 sufficiently provided an artificial co-stimulatory signal to enhance the antitumor immune response in a mouse model [65]. These authors engineered two versions of the dimeric anti-CD28 aptamer, one with a linker and one without, to precisely target the region of CD28 that could enhance the potency of the co-stimulatory signal. The dimeric version with a linker was designed by hybridization of two monomers with a previously reported 21-nucleotide structurally rigid DNA duplex, which reflects the average distance between two Fv of Ig molecules. The second version was directly engineered by combining two aptamers without any linker between the aptamers. Comparing the two versions, the dimeric aptamer construct without a linker has shown the strongest co-stimulatory capacity, much higher than the corresponding agonistic monoclonal antibody against CD28, while the dimeric aptamer with the linker showed similar proliferation ratio to an anti-CD28 antibody. In addition, the monomeric anti-CD28 aptamer blocked the binding of CD28′s main ligand, B7.2, leading to a strong inhibitory effect on the proliferation of purified CD4 lymphocytes. This early study demonstrated that aptamers could be engineered to perform as agonists or antagonists to induce cellular interactions, an impossible feat if corresponding antibodies are used [65].

**Figure 3:**
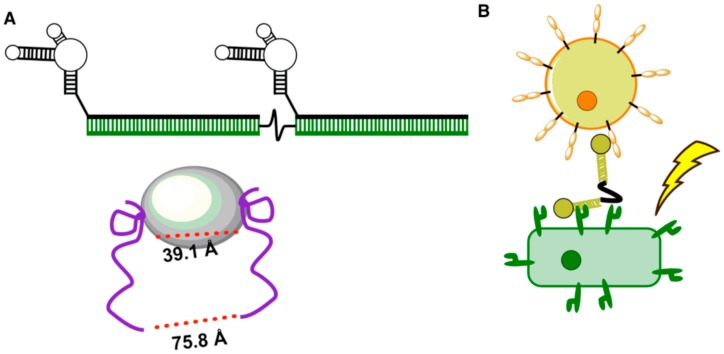
Engineered aptamers to modulate interactions in the immune system. (**A**) Bivalent aptamers were designed to modulate OX-40 receptors expressed in murine T cells. A polyethylene spacer was employed between the aptamers to enhance free rotation of the aptamers to enhance cross-linking [69]. The bivalent aptamers were designed to match the space between the two OX receptor′s natural ligand binding sites; (**B**) design of bispecific aptamers to modulate interactions in the immune system [68]. A number of bispecific aptamers were designed to direct immune cells towards cancer cells as potential immunotherapeutics [68].

To activate OX40, a member of the tumor necrosis factor receptor superfamily, a dimeric aptamer against OX40 was designed by exploiting the intrinsic ability of DNA to self-assemble [66]. It has been shown that OX40 receptor interacting with its ligand plays a major role in T cell proliferation and cytokine production. Thus, this study aimed to engineer an RNA aptamer against OX40 to stimulate OX40 function using murine models, followed by dimerizing the aptamer against OX40 with predetermined dimensions that precisely matched inter-OX40 distances to maximize receptor cross-linking [66]. Such scaffolds can only be constructed with building blocks that have precise measurements, such as DNA or RNA molecules, providing an elegant demonstration of the utility of DNA scaffolds combined with aptamer targeting moieties to engineer functional molecules. The engineered dimerized aptamer scaffold was able to activate the OX40 receptor and induce a cascade of biological interactions, enabling cytokine production that led to enhanced potency of a dendritic cell-based tumor vaccine in mice (Figure 3A) [66]. A multivalent aptamer-based DNA nanoconstruct was designed by Liu et al. to link two different cells [67]. Using a similar method, covalently linked, structurally diverse bispecific aptamer constructs were elegantly designed by Boltz et al. to investigate the potential of aptamers in developing immunotherapeutics by using aptamers against CD16α and cMyc expressed in breast cancer cells [68]. By designing 24 different bispecific aptamers in which all constructs were synthesized as single molecules, this study demonstrated how synthetic versatility of aptamers could be effectively utilized to fine-tune structural dimensions of a bispecific design. The most optimized bispecific design showed CD16α-mediated cell lysis induced by aptamer-directed cellular cytotoxicity (ADCC) with a magnitude similar to that of corresponding antibody (Figure 3B).

Furthermore, an engineered anti-PSMA aptamer has been reengineered as a bispecific aptamer, appending an anti-1BB aptamer. This bispecific aptamer effectively inhibited tumor growth and showed enhanced therapeutic index compared to antibodies, demonstrating the potential of engineered aptamers in developing effective molecules for immunotherapeutics [70].

An aptamer that targets mucin 1 was transformed into an effective imaging agent using ^99m^Tc [63]. Xion et al. demonstrated that lipid molecules tethered to a DNA aptamer could be utilized to effectively redirect immune cells towards target cells [71]. Utilizing an aptamer against 4-1BB on activated T cells, and McNamara et al. engineered multivalent anti-4-1BB aptamers to costimulate T cells [72]. An aptamer selected against CTLA-1, followed by multimeric assembly, termed Del 60 tetramer, was shown to inhibit the function of CTLA-4 with higher therapeutic index than that of monomeric Del 60 [69]. An aptamer against CD40 was developed by Soldevilla et al. and shown to work as an antagonist in its monomeric form, while the dimeric version showed agonistic behavior, demonstrating how engineering the aptamers into multimeric scaffolds could transform aptamer functionality [73].

### 3.3. Aptamers to Sense and Modulate Cell-Surface Interactions

**Figure 4:**
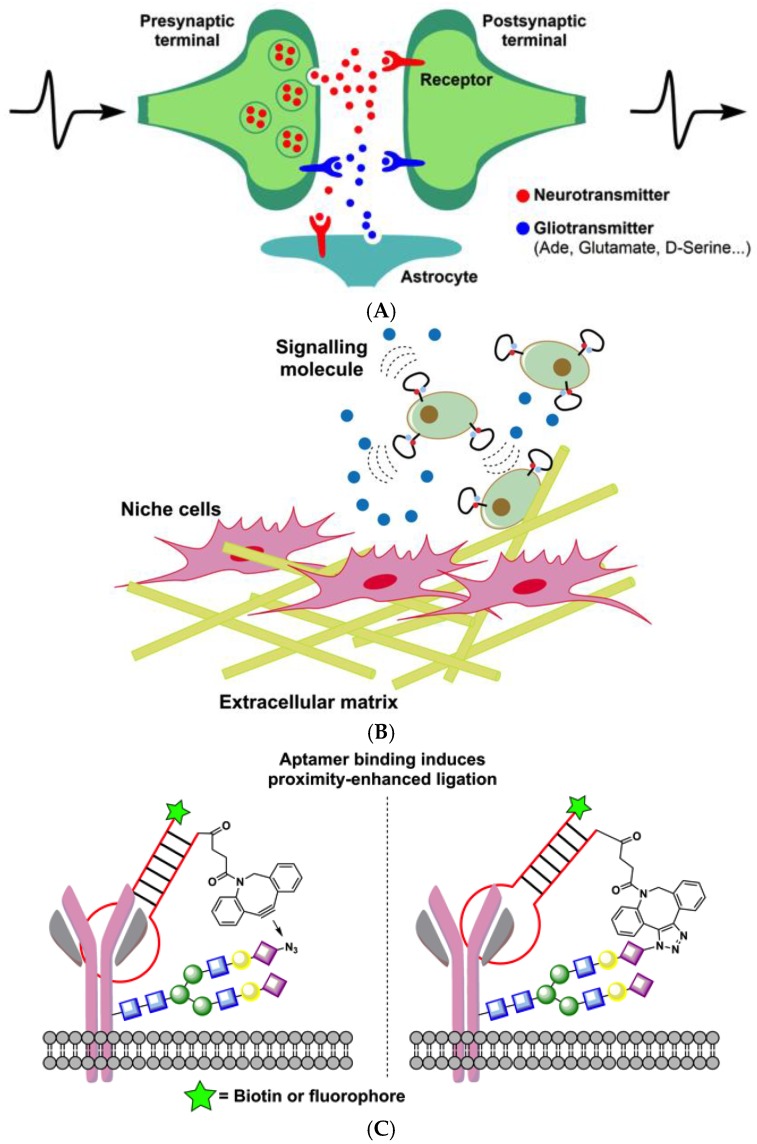
Aptamer-based molecular tools to probe cellular events. (**A**) Cell surface-anchored aptamer sensors to image chemical transmitter dynamics. Gliotransmitter adenine compounds in the form of ATP were detected using aptamer-based sensor [74]; (**B**) aptamer-based sensor immobilized on mesenchymal stem cell binding to probe signaling molecules secreted by niche cells [36]; (**C**) design of aptamer-based proximity ligation assays. Aptamer specific to cell-surface proteins utilized in detecting glycosylation patterns [75].

In addition to modulating molecular interactions in the immune system, aptamer-based molecular tools have been utilized to sense neuronal communication. For example, to sense the extracellular chemical transmission of dynamic events across the cell surface, Tokunaga et al. engineered a fast and responsive aptamer-based sensor to monitor in real-time the release of adenine compounds and, thus, serve as a gliotransmitter [74]. Specifically, in this study, authors demonstrate the application of an engineered aptamer with a fluorescent label against an adenine compound (as adenosine (ATP)) to sense extracellular adenine compounds. By directly anchoring a tocopherol-modified fluorescent aptamer, this study shows how aptamers could be utilized in real-time sensing of neurotransmitter release (Figure 4A). Zhao et al. engineered an aptamer-based sensor to probe cell signaling within the cellular niche environment [36]. In particular, an aptamer against PDGF was attached to the membrane of mesenchymal stem cells as a path toward understanding the unique biological processes in mesenchymal cells in their niche environment. An aptamer against PDGF was engineered with a pair of fluorescent dyes, and the conformation shift triggered by aptamer-target interaction led to a fluorescence signal allowing the spatiotemporal detection of PDGF secreted by cells within close proximity or added to the cellular environment. This study uniquely demonstrated how sensors could be developed by exploiting the conformational change that takes place when an aptamer binds its target PDGF, expanding the potential utility of aptamers in engineering sensors able to monitor events in real time on the cell surface (Figure 4B) [36]. Robinson et al. show how site-specific aptamers could be utilized in detecting protein glycol forms on live cell surfaces [75]. Using aptamer-cyclooctadiene (COD) conjugates, this report shows how to selectively ligate to an azido-sugar–labeled glycan exclusively expressed on target proteins on live cells [75]. In particular, this study uses two aptamers, one against PTK7 and another against membrane bound IgM. Aptamers are tethered to cyclooctadiene using a linker of optimal length against cells treated with *N*-azidoacetylmannosamine, showing that specific chemical ligation of the aptamer to glycosylated site could be achieved (Figure 4C). In a recent study, utilizing the same aptamers, You et al. demonstrated that aptamers binding to membrane proteins could be useful in studying transient membrane encounter rates [76]. These types of applications are more suited for aptamers, owing to their smaller size which leads to less impact on changing membrane’s physical structure, such as the rigidity, while, at the same time, providing information on unique interactions on the cellular membrane.

## 4. Methods of Aptamer Selection to against Cell Surface Receptors

We have examined how aptamers can be engineered to monitor and modulate the interactions of cell-bound protein receptors. However, the ultimate success of engineered molecules depends on specific targeting of the desired receptor. To accomplish this, many aptamer selection methods are available. Generally, aptamers are selected by a process called in vitro evolution of ligands by exponential enrichment (SELEX), which was introduced in the 1990s [77,78]. The core principle of SELEX is rooted in three interconnected steps, i.e., incubation, separation, and amplification [79]. Typically, aptamer molecules surviving the selection process are those with highest specificity and affinity to their cognate target. Many variations of the SELEX method have been introduced following its initial inception, but we will only focus on those SELEX methods that generate aptamers against specific cell-surface receptors expressed on whole cells.

### 4.1. Hybrid SELEX

Hybrid SELEX combines the strengths of SELEX against purified proteins with the use of whole cells to effectively identify high-affinity aptamers [80,81,82]. The strength of hybrid SELEX is rooted in the use of an already enriched pool against the exact protein receptor of interest, introducing a preconceived bias of the pool towards the target of interest, thus, avoiding potential interference with aptamers that can be enriched against potential co-receptors. Utilizing hybrid SELEX or reversed hybrid SELEX, a number of aptamers have been generated against key receptors [81,82]. For example, an anti-PSMA aptamer selected via hybrid SELEX has been utilized in drug, siRNA and nanoparticle delivery methods [83,84]. Additionally, an anti-transferrin aptamer was identified utilizing a slightly modified strategy called reversed crossover SELEX [82]. This aptamer was minimized and reengineered by appending it to a lipid moiety, which led to self-assembled liposomes to deliver siRNA to transferrin-positive Jurkat cells [73]. This strategy effectively demonstrated that aptamers could be engineered into functional nanomaterials and utilized as a delivery strategy. An anti-CD30 aptamer selected by hybrid SELEX, followed by truncation and reengineering into a trimeric and a dimeric aptamer, induced oligomerization of CD30 to activate downstream signaling events, eventually leading to apoptosis of anaplastic large cell lymphoma [85]. Using hybrid SELEX, a modified library of an extended genetic alphabet was recently used to identify aptamers against glypican 3, a potential marker in liver cancer cells using hybrid SELEX [86].

### 4.2. Live Whole Cell-SELEX

Live Whole cell-SELEX is a variant of complex target SELEX introduced by Larry Gold’s group [87,88,89]. Here, utilizing whole cells as the target and unrelated cells as a negative control, the method is exclusively designed to map out molecular signatures that exist between two cell types. This method shows the strength of SELEX in generating aptamers against membrane receptors in their native endogenous state [79]. This step represents significant progress given that many receptor proteins are difficult to purify or overexpress [79]. Furthermore, this method addressed the issue of protein misfolding and bias against different glycosylation patterns that appear on the protein owing to use of a bacterial expression system. Two aspects of live whole cell-SELEX have significantly enhanced the applicability of aptamers. First, whole cells and negative selection are used to identify biomarkers related to the positive cell line [90]. Second, flow cytometry is used as a detection tool to determine the progress of the selection using conditions similar to those of SELEX [79]. This is a significant deviation from original complex target SELEX method, which relied heavily on radioactive labeling and nitrocellulose filter methods, which might pose bias towards the aptamers identified and their affinity towards the target. Aptamers generated from live whole-cell SELEX have been used in a number of applications, including immunophenotyping, nanosensors, logic gates, and biomarker discovery [91,92,93,94,95].

### 4.3. Ligand-Guided Selection of Specific Aptamers

Recently, our group introduced a variant of complex target SELEX, termed Ligand-guided *S*election (LIGS) [96,97]. This is a variant of complex target SELEX that exploits the inherent nature of competition between weak and strong binders in a SELEX library. By exploiting the evolutionary nature of SELEX that leads to selection of aptamers, these aptamers are identified against precise sites of a cell-surface target guided by adding an external competitor [96,97]. Thus, one of the advantages of LIGS is the ability to select aptamers towards known cell surface targets at their native conformation using pre-existing receptor-ligand interactions. Such ligands can either simply outcompete aptamer candidates or they can induce a conformational switch to the target membrane receptor to destabilize the aptamer-receptor complex. Another advantage of LIGS is the ability to identify functional synthetic ligands against complex multidomain membrane proteins in their native environment. Given the complexity of these proteins, it is difficult to purify and mimic their actual structure in an artificial system, thus, challenging the identification of aptamers against these types of receptors. Aptamers generated against a specific target sensitive to receptor-ligand interactions can be utilized in engineering biosensors to detect these very interactions. On the other hand, LIGS is confined to selecting aptamers against membrane receptors that already have a naturally-existing or artificial ligand. However, given the wide range of potential modification that could be utilized in engineering aptamer-based molecular tools, identification of synthetic nucleic acid-based competing ligands against receptors would be advantageous. In addition, we have demonstrated that aptamers selected using LIGS could be further truncated to increase affinity towards their targets. Additionally, as demonstrated before, affinity could be further enhanced by effective and systematic truncation or multimerization strategies [98,99].

## 5. Conclusions

Aptamers were introduced in the 1990s and since then, many modifications to the SELEX method have resulted in improving the specificity, affinity, utility, and functionalization of aptamers, particularly for those selected against cell membrane proteins. Most recently, structural diversification of the library has been introduced, resulting in the enhancement of aptamers as potential therapeutic or diagnostic agents. The future of aptamers will increasingly involve logic gate-DNA complexes or engineering multivalent tools to tune host immune interactions to block pathogenic entry or replication or detect extracellular events as they occur in real-time on the cell membrane. It is safe to say that no other targeting molecule could perform these applications in a manner equal to aptamers and, as such, aptamer molecules will most certainly see an expanded role in biomedicine and bioassay in the coming years.
